# The American Journal of Insanity

**Published:** 1844-09

**Authors:** 


*The American Journal of Insanity.*—This is the title of a new periodical, the first No. of which was issued in July last, under the conduct of the officers of the New York State Lunatic Asylum, Utica. It is to be issued quarterly, at the exceedingly low price of $1.00 per annum, each No. containing 96 pages. We welcome with pleasure, this accession to the ranks of periodicals, the more that it fills a vacancy in medical literature, and advocates the cause of a large and pitiable class of sufferers. The No. before us; for which we are indebted to the courtesy of Dr. Brigham, Superintendent and Physician to the State Asylum; is fraught with valuable information, and contains several articles highly interesting to the general reader. If the publication sustains the promise given by its first number, it will hold a high place among medical periodicals.—[Ed.

